# Supplementary material for: Genome taxonomy of the genus Thalassotalea and proposal of Thalassotalea hakodatensis sp. nov. isolated from sea cucumber larvae
Source: PLoS One. 2023 Jun 2;18(6):e0286693. doi: 10.1371/journal.pone.0286693 (PMC10237450; doi:10.1371/journal.pone.0286693)
Supplement: S1 Table — (PDF) [file pone.0286693.s001.pdf]

Table S1. List of other *Colwelliaceae* genomes used for genome taxonomy of PTE2^T

Strains	Accession
Thalassomonas actinarium A5K-106^T	GCF_000948975.1
Thalassomonas virdans XOM25^T	GCF_000948985.1
Colwellia psychrerythraea 34H	GCF_000012325.1
Colwellia echini A3^T	GCF_002843355.2
Colwellia piezophila ATCC BAA-637^T	GCF_000378625.1
Colwellia demingiae ACAM 459^T	GCF_007954275.1
Colwellia polaris MCC 1C00015^T	GCF_002104515.1
Colwellia ponticola OISW-25^T	GCF_005885605.1
Colwellia chukchiensis CGMCC 1.9127^T	GCF_002104455.1
Colwellia hornerae ACAM 607^T	GCF_007954355.1
Colwellia marinimaniae MTCD1^T	GCF_002207865.1
Litorilituus sediminis JCM 17549^T	GCF_004295665.1
Litorilituus lipolyticus RZ04^T	GCF_006439335.1
Pseudocolwellia agarivorans QM50^T	GCF_002000085.1
Cognaticolwellia beringensis NB097-1^T	GCF_002076895.1
Cognaticolwellia mytili KCTC 52417^T	GCF_002104475.1
Cognaticolwellia aestuarii CGMCC1.6965	GCF_002104435.1
