# Supplementary material for: Genome taxonomy of the genus Thalassotalea and proposal of Thalassotalea hakodatensis sp. nov. isolated from sea cucumber larvae
Source: PLoS One. 2023 Jun 2;18(6):e0286693. doi: 10.1371/journal.pone.0286693 (PMC10237450; doi:10.1371/journal.pone.0286693)
Supplement: S2 Table — (PDF) [file pone.0286693.s002.pdf]

Table S2. ***In silico*** **DDH and ANI values of** ***Thalassotalea hakodatensis*** **PTE2^T sp. nov. against** ***Thalassotalea*** **species**

Species	*in silico* DDH	ANI
Thalassotalea sediminis KCTC 42588^T	20.8%	77.1%
Thalassotaleainsulae KCTC 62186^T	20.1%	72.0%
Thalassotalea piscium JCM 18590^T	22.0%	71.9%
Thalassotalea agarivorans JCM 13379^T	24.6%	70.1%
Thalassotalea loyana LMG 22536^T	21.8%	70.5%
Thalassotalea eurytherma JCM 18482^T	21.6%	70.6%
Thalassotalea atypica JCM 31894^T	23.1%	70.7%
Thalassotalea marina QBLM2^T	19.7%	72.5%
Thalassotalea profundi YM155^T	20.4%	71.7%
Thalassotalea mangrovi zs-4^T	23.4%	68.5%
Thalassotalea crassostreae LPB0090^T	24.3%	69.6%
Thalassotalea algicola M1531^T	21.0%	70.9%
Thalassotalea litorea MCCC IK03283	24.7%	68.9%
Thalassotalea euphylliae H2	22.8%	70.9%
