# Supplementary material for: Genome taxonomy of the genus Thalassotalea and proposal of Thalassotalea hakodatensis sp. nov. isolated from sea cucumber larvae
Source: PLoS One. 2023 Jun 2;18(6):e0286693. doi: 10.1371/journal.pone.0286693 (PMC10237450; doi:10.1371/journal.pone.0286693)
Supplement: S3 Table — Strains: 1, T. sediminis KCTC 42588 T, 2, T. insulae KCTC 42588 T, 3, T. piscium JCM 18590 T, 4, T. marina KCTC 42731T, 5, T. profundi YM155T, 6, T. agarivorans JCM 13379 T, 7, T. eurytherma JCM 18482T, 8, T. atypica JCM 31894 T, 9, T. mangrovi zs-4T, 10, T. crassostreae LPB 0090T,11, T. loyana LMG 22536T, 12, T. algicola M1531T, 13, T. litorea HMF4135T, 14, T. euphylliae Eup-16T. (PDF) [file pone.0286693.s003.pdf]

Table S3. Fatty acid, isoprenoid quinone and polar lipid profile of previously reported *Thalassotalea*

	1	2	3	4	5	6	7	8	9	10	11	12	13	14
**Predominant FA**														
**C9:0**	TR	TR	TR	1.4	TR	TR	-	-	-	TR	-	-	-	-
**C10:0**	2.3	2.6	1.8	4.9	1.4	1.1	TR	TR	TR	-	-	-	1.3	-
**C11:0**	TR	TR	TR	TR	TR	2.2	TR	-	1.6	TR	-			
**C12:0**	-	TR	TR	TR	TR	4.8	2.2	1.7	-	-	-	-	2.9	2.1
**C13:0**	-	-	TR	TR	-	7.3	1.6	TR	4.7	4.7	-	-	TR	-
**C14:0**	6.9	7.1	2.2	3.7	TR	4.7	9.9	5.1	5.9	4.5	13	-	2.6	1.7
**C15:0**	-	-	-	-	-	-	5.7	-	-	-	-	-	-	-
**C16:0**	10.8	16.8	19.5	7.8	5.2	17.5	9.4	18.5	15	22.7	5	-	17.9	15.3
**C17:0**	2.6	TR	3	1.8	1.9	11.1	1	1.2	3.6	5.5	-	-	1.9	4.1
**C18:0**	-	-	-	-	-	-	-	-	-	-	-	-	-	4.3
**Branched:**														
**iso-C14:0**	1	1.3	TR	2.5	1.8	4.7	-	-	2.4	-	-	-	TR	-
**iso-C16:0**	2.1	5.4	3.4	7.2	14.6	TR	-	TR	2.4	0.7	-	-	1	-
**Unsaturated:**														
**C15:1 ω8c**	6.8	3.2	4.1	2.8	4.9	2.7	4.8	4.9	7.3	-	-	-	1.5	5.4
**C16:1 ω9c**	3.6	5.1	10.1	2.7	3.5	-	4.5	4.9	5.7	-	5.7	-	11.5	1.8
**iso-C17:1 ω5c**	-	-	-	-	-	-	0.3	9.5	-	-	-	-	-	-
**C17:1 ω8c**	20	7.4	9	20.6	25.1	12.8	14.9	9.5	24.2	15.4	12	-	8.8	12.5
**C18:1 ω7c**	1.7	2.9	2	2.6	1.7	4.8	3.4	3.3	-	-	-	-	9.1	15.7
**Hydroxy:**														
**C10:0 3-OH**														1.4
**C11:0 3-OH**	3.5	1.6	1.1	5.4	2.5	1.4	3.7	2.1	2.2	-	-	-	1.6	2.6
**C12:0 3-OH**	2.9	3.3	3.8	5.4	1.4	2.2	-	3.9	1.1	0.4	-	-	10.2	3.8
**iso-C12:0 3-OH**	2.1	3	3	-	6	-	-	-	TR	-	-	-	1.8	-
**Summed features:**														
**3 (C16:1 ω7c and/or C16:1 ω6c)**	15.6	32.7	23.4	13.3	16.3	8.6	25.1	36	9.1	17.3	31.3	major	16.9	25.5
**8 (C18:1 ω7c and/or C18:1 ω6c)**	-	-	-	-	-	-	-	-	-	4.6	-	major	4.8	-
**Major lipid class**														
**PG**	+	+	+	+	+	nd	nd	+	+	+	nd	+	+	+
**PE**	+	+	+	+	+	nd	nd	+	+	+	nd	+	+	+
**Isoprenoid quinones**	Q-8	Q-8	Q-8	Q-8	Q-8	nd	Q-8	Q-8	Q-8	Q-8	nd	Q-8	Q-8	Q-8
**References**	[8]	[16]	[1]	[7]	[13]	[4]	[19]	[15]	[17]	[11]	[3]	[18]	[12]	[9]

Strains: 1, *T. sediminis* KCTC 42588 ^{T}, 2, *T. insulae* KCTC 42588 ^{T}, 3, *T. piscium* JCM 18590 ^{T}, 4, *T. marina* KCTC 42731^{T}, 5, *T. profundi* YM155^{T}, 6, *T. agarivorans* JCM 13379 ^{T}, 7, *T. eurytherma* JCM 18482^{T}, 8, *T. atypica* JCM 31894 ^{T}, 9, *T. mangrovi* zs-4^{T}, 10, *T. crassostreae* LPB 0090^{T},11, *T. loyana* LMG 22536^{T}, 12, *T. algicola* M1531^{T}, 13, *T. litorea* HMF4135^{T}, 14, *T. euphylliae* Eup-16^{T}.
