# Supplementary material for: Genome taxonomy of the genus Thalassotalea and proposal of Thalassotalea hakodatensis sp. nov. isolated from sea cucumber larvae
Source: PLoS One. 2023 Jun 2;18(6):e0286693. doi: 10.1371/journal.pone.0286693 (PMC10237450; doi:10.1371/journal.pone.0286693)
Supplement: S4 Table — (PDF) [file pone.0286693.s004.pdf]

**Table S4. FAS associated genes composition of 15 species**

| Gene names | *T. hakodatensis* sp. nov. PTE2$^{T}$ | *T. sediminis* | *T. insulae* | *T. piscium* | *T. marina* | *T. profundi* | *T. agarivorans* | *T. eurytherma* | *T. atypica* | *T. mangrovi* | *T. crassostreae* | *T. loyana* | *T. algicola* | *T. litorea* | *T. euphylliae* |
|---|---|---|---|---|---|---|---|---|---|---|---|---|---|---|---|
| *accA* | + | + | + | + | + | + | + | + | + | + | + | + | + | + | + |
| *accB* | + | + | + | + | + | + | + | + | + | + | + | + | + | + | + |
| *accC* | + | + | + | + | + | + | + | + | + | + | + | + | + | + | + |
| *accD* | + | + | + | + | + | + | + | + | + | + | + | + | + | + | + |
| *fabD* | + | + | + | + | + | + | + | + | + | + | + | + | + | + | + |
| *fabH* | + | + | + | + | + | + | - | + | + | + | + | + | + | + | + |
| *fabY* | + | + | + | + | + | + | + | - | - | - | - | - | - | - | - |
| *fabB* | + | + | + | + | + | + | + | + | + | + | + | + | + | + | + |
| *fabF* | + | + | + | + | + | + | + | + | + | + | + | + | + | + | + |
| *fabG* | + | + | + | + | + | + | + | + | + | + | + | + | + | + | + |
| *fabA* | + | + | + | + | + | + | + | + | + | + | + | + | + | + | + |
| *fabZ* | + | + | + | + | + | + | + | + | + | + | + | + | + | + | + |
| *fabV* | + | + | + | + | + | + | + | + | + | + | + | + | + | + | + |
| *lpxA* | + | + | + | + | + | + | + | + | + | + | + | + | + | + | + |
| *lpxD* | + | + | + | + | + | + | + | + | + | + | + | + | + | + | + |
| *ilvE* | + | + | + | + | + | + | + | + | + | + | + | + | + | + | + |
| *dkdA* 1 | + | + | + | + | + | + | + | + | + | + | + | + | + | + | + |
| *dkdA* 2 | + | + | + | + | + | + | + | + | + | + | + | + | + | + | + |
| *dkdB* | + | + | + | + | + | + | + | + | + | + | + | + | + | + | + |
| *Des1* | + | + | + | + | + | + | + | + | + | + | + | + | + | + | + |
| *fadB* | + | + | + | + | + | + | + | + | + | + | + | + | + | + | + |
| *fadJ* | + | + | + | + | + | + | + | + | + | + | + | + | + | + | + |
| *acuI* | + | + | + | + | + | + | - | - | - | - | + | + | + | - | + |
