# Supplementary material for: Genome taxonomy of the genus Thalassotalea and proposal of Thalassotalea hakodatensis sp. nov. isolated from sea cucumber larvae
Source: PLoS One. 2023 Jun 2;18(6):e0286693. doi: 10.1371/journal.pone.0286693 (PMC10237450; doi:10.1371/journal.pone.0286693)
Supplement: S5 Table — (PDF) [file pone.0286693.s005.pdf]

Table S5. Results of 3D-structure prediction of Des1 by Phyre2

Species	PDB Molecule	Template	Confidence	Coverage	Identity
Thalassotalea hakodatensis sp. nov. PTE2^{T}	Stearoyl-CoA desaturase	c4zyoA	100.0%	72%	30%
Thalassotalea sediminis KCTC 42588^{T}	Stearoyl-CoA desaturase	c4ymkA	100.0%	76%	30%
Thalassotalea insulae KCTC 62186^{T}	Stearoyl-CoA desaturase	c4ymkA	100.0%	76%	30%
Thalassotalea piscium JCM 18590^{T}	Stearoyl-CoA desaturase	c4ymkA	100.0%	76%	30%
Thalassotalea agarivorans JCM 13379^{T}	Stearoyl-CoA desaturase	c4ymkA	100.0%	75%	30%
Thalassotalea loyana LMG 22536^{T}	Stearoyl-CoA desaturase	c4ymkA	100.0%	76%	27%
Thalassotalea eurytherma JCM 18482^{T}	Stearoyl-CoA desaturase	c4ymkA	100.0%	75%	27%
Thalassotalea atypica JCM 31894^{T}	Stearoyl-CoA desaturase	c4ymkA	100.0%	76%	29%
Thalassotalea marina QBLM2^{T}	Stearoyl-CoA desaturase	c4ymkA	100.0%	77%	29%
Thalassotalea profundi YM155^{T}	Stearoyl-CoA desaturase	c4ymkA	100.0%	76%	31%
Thalassotalea mangrovi zs-4^{T}	Stearoyl-CoA desaturase	c4ymkA	100.0%	80%	30%
Thalassotalea crassostreae LPB0090^{T}	Stearoyl-CoA desaturase	c4ymkA	100.0%	74%	32%
Thalassotalea algicola M1531^{T}	Stearoyl-CoA desaturase	c4ymkA	100.0%	76%	30%
Thalassotalea litorea MCCC IK03283	Stearoyl-CoA desaturase	c4zyoA	100.0%	75%	30%
Thalassotalea euphylliae H2	Stearoyl-CoA desaturase	c4ymkA	100.0%	76%	28%
