# Supplementary material for: Genome taxonomy of the genus Thalassotalea and proposal of Thalassotalea hakodatensis sp. nov. isolated from sea cucumber larvae
Source: PLoS One. 2023 Jun 2;18(6):e0286693. doi: 10.1371/journal.pone.0286693 (PMC10237450; doi:10.1371/journal.pone.0286693)
Supplement: S6 Table — (PDF) [file pone.0286693.s006.pdf]

Table S6. PG, PE and DPG associated genes composition of each strain

Gene names	*T. hakodatensis* sp. nov. PTE2[T]	*T. sediminis*	*T. insulae*	*T. piscium*	*T. marina*	*T. profundi*	*T. agarivorans*	*T. eurytherma*	*T. atypica*	*T. mangrovi*	*T. crassostreae*	*T. loyana*	*T. algicola*	*T. litorea*	*T. euphylliae*
plsX	+	+	+	+	+	+	+	+	+	+	+	+	+	+	+
plsY	+	+	+	+	+	+	+	+	+	+	+	+	+	+	+
plsC	+	+	+	+	+	+	+	+	+	+	+	+	+	+	+
cdsA	+	+	+	+	+	+	+	+	+	+	+	+	+	+	+
pssA	+	+	+	+	+	+	+	+	+	+	+	+	+	+	+
psd	+	+	+	+	+	+	+	+	+	+	+	+	+	+	+
pgsA	+	+	+	+	+	+	+	+	+	+	+	+	+	+	+
pgpA	+	+	+	+	+	+	+	+	+	+	+	+	+	+	+
clsA/B	-	-	-	-	-	-	-	-	-	+	-	-	-	+	-
clsC	-	-	-	+	-	-	+	-	-	-	+	-	-	+	-
