# Supplementary material for: Genome taxonomy of the genus Thalassotalea and proposal of Thalassotalea hakodatensis sp. nov. isolated from sea cucumber larvae
Source: PLoS One. 2023 Jun 2;18(6):e0286693. doi: 10.1371/journal.pone.0286693 (PMC10237450; doi:10.1371/journal.pone.0286693)
Supplement: S1 Fig — ACP: acyl-carrier protein; AccABCD: acetyl-CoA carboxylase complex; FabD: malonyl-CoA: ACP transacylase; FabH/FabY: 3-ketoacyl-ACP synthase Ⅲ; FabB: 3-ketoacyl-ACP synthase Ⅰ; FabF: 3-ketoacyl-ACP synthase Ⅱ; FabG: 3-ketoacyl-ACP reductase; FabA: 3-hydroxyacyl-ACP dehydratase/trans-2-decenoyl-ACP isomerase; FabV enoyl-ACP reductase; FabZ: 3-hydroxyacyl-ACP dehydratase. (PDF) [file pone.0286693.s007.pdf]

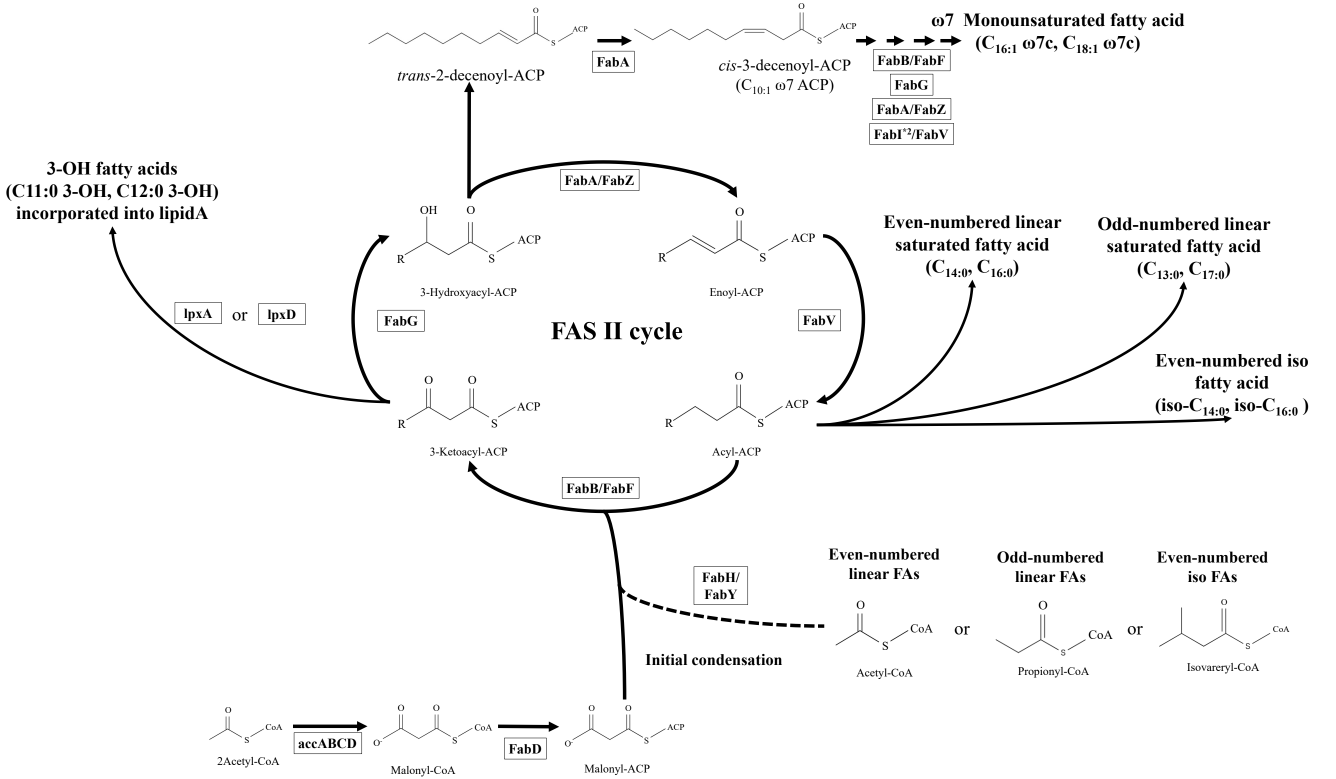

Fig S1. Predicted fatty acid synthetic pathway in *Thalassotalea* species.

ACP: acyl-carrier protein; AccABCD: acetyl-CoA carboxylase complex; FabD: malonyl-CoA: ACP transacylase; FabH/FabY: 3-ketoacyl-ACP synthase III; FabB: 3-ketoacyl-ACP synthase I; FabF: 3-ketoacyl-ACP synthase II; FabG: 3-ketoacyl-ACP reductase; FabA: 3-hydroxyacyl-ACP dehydratase/trans-2-decenoyl-ACP isomerase; FabV enoyl-ACP reductase; FabZ: 3-hydroxyacyl-ACP dehydratase.
