# Supplementary material for: Genome taxonomy of the genus Thalassotalea and proposal of Thalassotalea hakodatensis sp. nov. isolated from sea cucumber larvae
Source: PLoS One. 2023 Jun 2;18(6):e0286693. doi: 10.1371/journal.pone.0286693 (PMC10237450; doi:10.1371/journal.pone.0286693)
Supplement: S3 Fig — Protein/enzyme name each gene is coding: fabA: 3-hydroxyacyl-ACP dehydrase/trans-2-decenoyl-ACP isomerase; fabD: malonyl-CoA: ACP transacylase; fabF: 3-ketoacyl-ACP synthase Ⅱ; fabG: 3-ketoacyl-ACP reductase; fabH: 3-ketoacyl-ACP synthase Ⅲ; fabV enoyl-ACP reductase; fabY: 3-ketoacyl-ACP synthase; fabZ: 3-hydroxyacyl-ACP dehydratase; accABCD: carboxylase complex; plsX: phosphate acyltransferase; acpP: Acyl-carrier protein. (PDF) [file pone.0286693.s009.pdf]

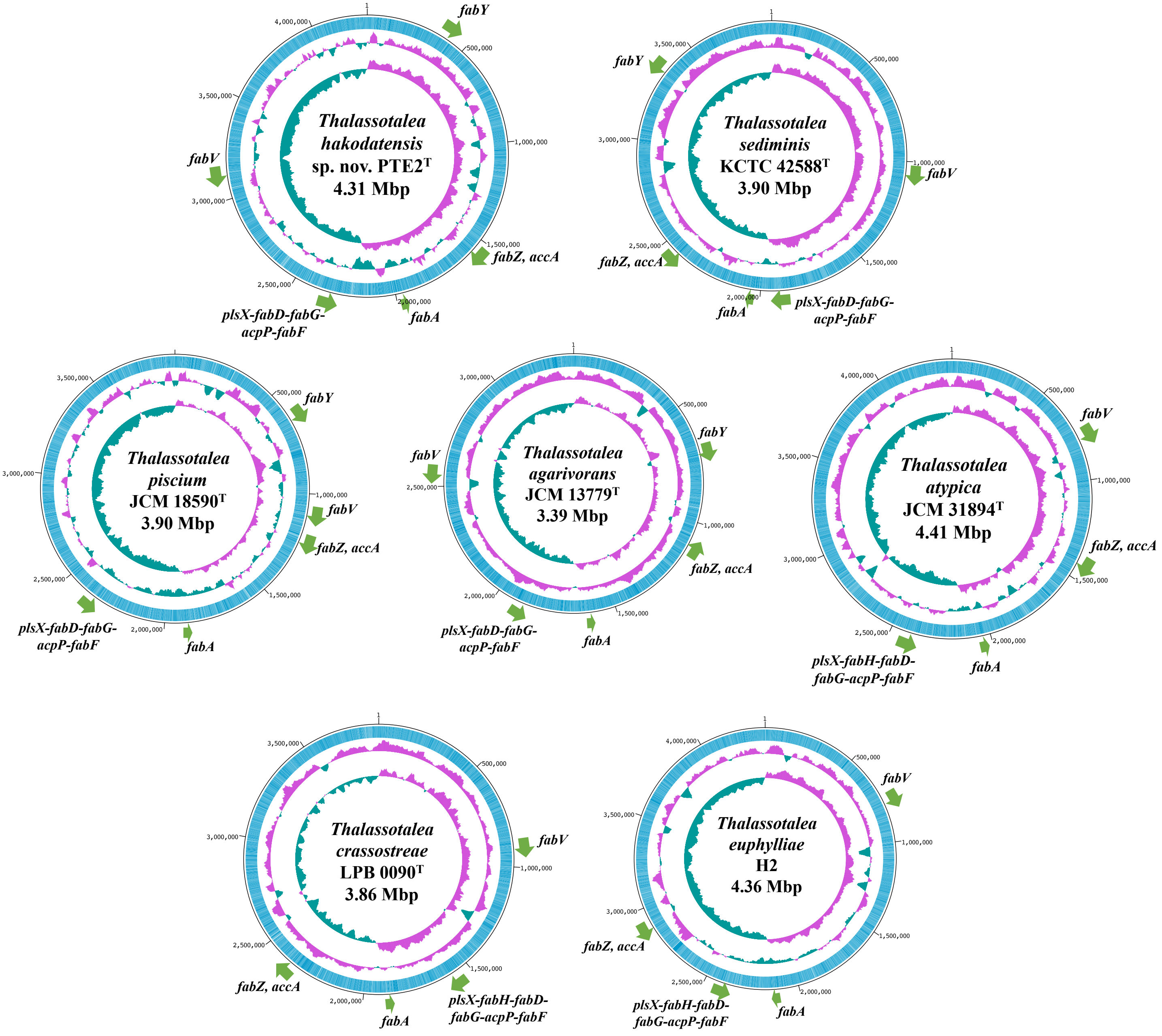

Fig S3. Genomic distribution of *fab* and associated genes (only strains with complete genome sequence).

Protein/enzyme name each gene is coding: *fabA*: 3-hydroxyacyl-ACP dehydrase/trans-2-decenoyl-ACP isomerase; *fabD*: malonyl-CoA: ACP transacylase; *fabF*: 3-ketoacyl-ACP synthase II ; *fabG*: 3-ketoacyl-ACP reductase; *fabH*: 3-ketoacyl-ACP synthase III; *fabV* enoyl-ACP reductase; *fabY*: 3-ketoacyl-ACP synthase; *fabZ*: 3-hydroxyacyl-ACP dehydratase; *accABCD*: carboxylase complex; *plsX*: phosphate acyltransferase; *acpP*: Acyl-carrier protein.
