# Supplementary material for: Genome taxonomy of the genus Thalassotalea and proposal of Thalassotalea hakodatensis sp. nov. isolated from sea cucumber larvae
Source: PLoS One. 2023 Jun 2;18(6):e0286693. doi: 10.1371/journal.pone.0286693 (PMC10237450; doi:10.1371/journal.pone.0286693)
Supplement: Attachment — Submitted filename: Response_letter_30Apr23.docx [file pone.0286693.s010.docx]

Dear Professor Thompson, the editor, and reviewers,

We appreciate editor and reviewers for constructive suggestions for PONE-D-23-07380. We improved the manuscript according to the editorial office and reviewers’ comments. Responses for specific comments are described as follows. All changes were highlighted in yellow.

Editorial office

1. Please ensure that your manuscript meets PLOS ONE's style requirements, including those for file naming. The PLOS ONE style templates.

Response: Thank you so much for careful check. Style was fit to PLOS ONE format.

2. We noticed you have some minor occurrence of overlapping text with the following previous publication(s), which needs to be addressed.

Response: Thank you for the suggestion. We used almost same approach, so many parts of body text were overlapped. We did remove redundancy and put the previous paper as reference.

Reviewer: 1

1. The authors present a sound body of evidence to justify the description of this new species of the genus Thalassotalea. It is a good example of genomic taxonomy. I am convinced that this format is adequate for the description of a new species.

Response: Thank you so much for the positive comments.

Reviewer: 2

1. The study was well conducted, and the results are very interesting. Congratulations. However, I have some points that need to be addressed. First, please check the figure´s quality.

Response: Thank you for the comments. We tried to increase figs resolution.

2. "suggesting strain PTE2 may pale an important role in host-microbe interaction during sea cucumber development" Did the authors mean "may play"?

Response: Thank you so much for the suggestion. We did correct it.

3. The 16S sequence was generated using which technology? How these sequences were processed? Please clarify.

Response: Thank you for the comments. We put the methodology.

4. It is not clear what genomes were used in OrthoANI and DDH analysis.

Response: Thank you for the comments. We put the option description; calculate OrthoANI with a default setting and DDH by formula 2.

5. Do you know if the Figure 1 subtitle is correct? Because Idiomarina rambicola is not visible in the figure.

Response: Idiomarina was used for outgroup, but after figuring rooted subtree shown in Fig 1, Idiomarina position was not visible. We added some explanation in Fig 1 title.

6. What were the parameters used in MLSA phylogeny? ML? NJ? Bootstrap?

How did the MLSA network build? I could not find the section in Methodology.

Response: Thank you for the comments. We also put some Idiomarina sequences in Fig 3, and options for MLSA.

6. I suggest that the phrase below could be written removing "I was not able". "However, I was not able to identify a pathway to produce some of the major fatty acids such as C15:1 ω8c and iso-C17:1 ω5C."

Response: Thank you for the comments. According to the suggestion, we did correct the sentence.
